# An Interactive Optimal Scheduling Method for Hydrogen Production System with Heat Recovery

**DOI:** 10.3390/e28020194

**Published:** 2026-02-09

**Authors:** Shengchen Li, Wenbin Wu, Zhenhang Wu, Linrui Ma, Yang Si

**Affiliations:** 1 Qinghai Key Lab of Efficient Utilization of Clean Energy, School of Energy and Electrical Engineering, University of Qinghai, Xining 810016, China; 2 Engineer School, Qinghai Institute of Technology, Xining 810016, China; 3 Hubei Energy Group Ezhou Power Generation Co., Ltd., Ezhou 436032, China

**Keywords:** alkaline electrolyser, renewable energy hydrogen production, waste heat recovery, interactive optimisation, multi-condition operation, economic analysis

## Abstract

Renewable intermittency forces electrolytic hydrogen systems to operate across multiple states, lowering efficiency. We design a thermodynamic cycle that recovers electrolysis waste heat and integrates it with an alkaline electrolyser. A detailed thermodynamic model of the hydrogen system and the heat-recovery loop is developed, and design and operating parameters are optimized to maximize overall exergy efficiency. To improve economic viability, heat-exchanger structural parameters are co-optimized. We further propose an optimal scheduling method for the heat-recovery system under fluctuating renewable supply. The method employs an interactive optimisation framework cantered on the temperature–efficiency curve of alkaline electrolyser cells, jointly optimizing electrolyser current and working-fluid mass flow to enhance economic performance. A case study using real wind-farm data from Qinghai demonstrates that the proposed system with heat recovery significantly improves performance, increasing hydrogen production by up to 9% under wind scarcity compared to that of the system without heat recovery. These results confirm the practical viability of renewable-driven hydrogen production.

## 1. Introduction

Fossil fuels account for approximately 80% of the global energy supply [1]. The current fossil-fuel-dominated energy structure, coupled with intensifying climate concerns, necessitates a rapid expansion of renewable energy systems as a fundamental approach to sustainable energy security. In sectors requiring high-temperature thermal energy, such as transportation and heavy industry, decarbonisation cannot be achieved solely through electrification [2]. Therefore, it is necessary to find cleaner energy sources for comprehensive decarbonisation. Hydrogen, with its dual material and energy characteristics, offers substantial potential for global energy decarbonisation.

Currently, 76% of the hydrogen produced in the industry is derived from natural gas (grey hydrogen), with the remaining 23% originating from coal (black hydrogen). Environmental emissions from hydrogen production processes significantly undermine the environmental benefits of hydrogen as a source of energy. Hydrogen production through water electrolysis using renewable energy (green hydrogen), with its clean and pollution-free characteristics, has become a growing research focus in the hydrogen industry. The existing electrolytic hydrogen production technologies mainly include hydrogen production by electrolysing water in alkaline electrolyser cells (AECs) [3,4], solid oxide electrolyser cells (SOEC) [5,6], and proton exchange membrane electrolyser cells (PMEC) [7,8]. Among the three electrolyser technologies, alkaline electrolysis cells (AECs) demonstrate superior suitability for large-scale deployment due to their cost-effectiveness and technological maturity. Although electrolysis of water for hydrogen production addresses the cleanliness and sustainability of hydrogen sources, green hydrogen still struggles to compete favourably with grey and black hydrogen due to its higher cost. To enhance the competitiveness of green hydrogen, it is essential to optimise the operating strategy of the hydrogen production system to improve overall system efficiency and reduce hydrogen costs.

The economic performance of the hydrogen production system is primarily determined by the hydrogen production volume, which is influenced mainly by the production efficiency of alkaline electrolysis cells (AECs). Currently, there are two modelling approaches regarding the relationship between the hydrogen production efficiency and the electrical energy input to the electrolyser. One is that the hydrogen production efficiency is positively correlated with the input electrical energy, meaning that the hydrogen production rate is a constant value. The linear modelling approach assumes constant hydrogen production rates, with efficiency directly proportional to the electrical input. Studies [9,10,11] demonstrate this approach through various applications: low-carbon dispatch for high-renewable multi-energy systems with optimised reserve capacity [9], integrated demand response strategies for electricity–hydrogen systems with energy complementarity frameworks [10], and power-to-gas microgrid optimisation, minimising operational costs while satisfying energy demands and safety constraints [11].

However, electrolytic hydrogen production involves multiple interdependent factors and reaction mechanisms, such as current density, electrolyte composition, temperature, and pressure. The interactions among these factors are usually not simple linear relationships. Therefore, the interactions among these variables typically exhibit nonlinear characteristics. Consequently, nonlinear models provide a superior representation of these complex interactions, thereby improving model accuracy and predictive capability. The literature [12] constructs a nonlinear hydrogen production model of AECs, proposes a virtual power plant model of wind–solar–hydrogen–thermal power based on the wide power adaptation model of AECs and uses an improved multi-path meshless ray optimisation algorithm for equipment output scheduling and capacity configuration optimisation. The simulation results show that this model not only reduces the operating cost of the system but also effectively copes with the fluctuations in wind and solar power output, thereby enhancing the accommodation level of wind and solar energy. Reference [13] utilises AECs as the power-to-hydrogen equipment, examines its nonlinear hydrogen production efficiency curve, and develops an optimisation configuration model that integrates operation interval planning and economic planning. Through the chaotic particle swarm optimisation algorithm, it effectively enhances system operation reliability, reduces investment costs and the wind power curtailment rate, and enables the flexible tracking of the power-to-hydrogen equipment to power fluctuations in renewable energy.

The referenced studies demonstrate nonlinear electrolyser efficiency, modelling that effectively leverages operational flexibility across diverse conditions, thereby enhancing the integration of renewable energy. However, renewable energy intermittency and safe operating constraints necessitate electrolyser shut-down during insufficient wind and solar output, accompanied by thermal losses. Upon energy recovery, electrolysers require preliminary heating before resuming production, as operating temperatures fall below electrolysis thresholds during shut-down periods [14,15]. This restart process inherently reduces system efficiency. Consequently, researchers have developed multi-state operational models that incorporate hydrogen production, thermal standby, and shut-down phases to address these thermal dynamics. Therefore, some scholars have proposed modelling schemes that consider the multi-state operation scenarios of electrolysers, including the hydrogen production stage, thermal standby stage, and shut-down stage. The literature [16] established a nonlinear model of hydrogen production efficiency, considering the economic model of three operating states of the electrolyser for the capacity configuration of multiple operating states in the power-to-hydrogen system. On this basis, the optimal capacity configuration problem of the electrolyser was integrated into a mixed-integer nonlinear programming problem and compared with the two-state simple model. The results show that the electrolyser model, which considers multiple operating states, can reduce the cost of hydrogen production. The literature [17] established the optimal dispatch model of the alkaline electrolyser as a mixed-integer linear problem. By solving this problem, the number of electrolysers was optimised, and the optimal installation number of the electrolysers corresponded to 54% of the number of electrolysers required to absorb the highest energy peak.

While the referenced studies develop nonlinear efficiency models incorporating multi-state electrolyser operations, they fail to account for thermal dynamics. Temperature degradation occurs during thermal standby and shut-down phases, significantly affecting electrolyte conductivity, catalyst activity, and bubble coverage phenomena. Consequently, temperature effects on electrolysis efficiency represent a critical modelling parameter that cannot be disregarded. Building on these limitations, Reference [18] proposed a two-stage electrolyser hydrogen production model that considers start-up and shut-down states while establishing the electrolyser temperature model. It used this model to propose a planning model for the integrated electricity–hydrogen energy system. The thermal cycle of the electrolytic hydrogen production system is detailed in the paper; however, it inadequately addresses the interrelationship between hydrogen production, electrical input, and temperature and fails to accurately model daily operational state transitions.

Reference [19] established a nonlinear model of hydrogen production efficiency and a multi-state operation model of the electrolyser, considering the use of the lumped capacitance method to develop a temperature model of the alkaline electrolyser. The established model can describe the hydrogen production characteristics, temperature changes and state changes of the alkaline electrolyser. When applied to economic optimal dispatch strategies considering time-of-use electricity pricing, the model demonstrated a significant enhancement in the profitability of the hydrogen production system.

In practice, electrolytic reactions generate substantial heat during operation. To maintain the optimal temperature of the electrolytic cell, the hydrogen production system requires an external condenser to cool the circulating electrolytes, resulting in significant energy consumption. Reference [20] thus established a waste heat recovery model based on principles of heat transfer and thermodynamics. It was found that the recovered waste heat comprises 30 times the energy needed to heat the electrolysed water. Using the recovered heat to heat the electrolysed water increased the efficiency of the hydrogen production system by 0.33% and the hydrogen output by 0.22%. Reference [21] developed a nonlinear hydrogen production model and a heat recovery model for alkaline electrolytic cells, utilising waste heat to supply distributed heating systems. It also proposed a daily optimal operation strategy for multi-energy systems based on economic model predictive control, which achieves economically optimal operation through optimal scheduling of various distributed energy sources. The results show that compared with the rule-based strategy and the scenario without the electro–hydrogen–thermal model, the operation costs were reduced by 59% and 38%, respectively.

Reference [22] develops a dynamic electrolyser-efficiency model that jointly accounts for load variations, start-up/shut-down cycling, and thermal management. It further employs a rolling-horizon optimisation strategy to smooth the load profile, stabilize operating temperatures, and increase hydrogen production. Reference [23] formulates a multi-objective, constrained operational optimisation model for a wind-battery storage–alkaline electrolyser system, balancing profit maximization against power curtailment minimization while incorporating practical constraints such as minimum start-up/shut-down times, upper and lower power limits, and input fluctuation bounds. In contrast, this paper proposes an interactive optimisation framework that bidirectionally couples a detailed electro-thermal electrolyser model with a process-level heat-recovery loop model through an iterative coordination scheme. This framework enables a quantitative evaluation of how different heat-recovery integration schemes affect dynamic efficiency and overall system economics under wind power fluctuations.

The aforementioned studies have also developed nonlinear hydrogen production efficiency models and investigated the recovery of electrolyser waste heat for in-system utilization and distributed heating to improve economic performance. However, when heat recovery is considered, a rigorous process-level heat-recovery model is not always established. Moreover, this paper posits that using waste heat solely to preheat the feedwater typically yields only marginal gains in overall system efficiency, and these gains diminish further as electrolyser capacity increases. Therefore, merely preheating the feedwater with waste heat is unlikely to significantly enhance system efficiency. By contrast, we hypothesize that utilizing waste heat during the electrolyser shut-down phase to maintain its temperature—thereby shortening subsequent start-up time—can more effectively support increased hydrogen production.

Incorporating a heat-recovery cycle transforms an originally straightforward hydrogen production system into a substantially more complex configuration. The introduction of multiple nonlinear components and diverse operating modes across varying working conditions markedly intensifies the system’s nonlinear behaviour. Based on this complexity, we argue that conventional solution methods often struggle to identify the global optimum in such settings, which can prevent the system from fully realizing its potential in practice. To address this challenge, this paper proposes an interactive optimisation approach that establishes an explicit interaction mechanism between the heat-recovery system and the hydrogen production system and evaluates the proposed method under different wind turbine output scenarios.

## 2. Hydrogen Production System with Heat Recovery

The frequent start-up and shut-down of AECs under wind, solar, and other fluctuating power sources, as well as the unsteady operation process, will have adverse effects on hydrogen production. AECs may be accompanied by multi-state operation under a fluctuating power supply. From the perspective of waste heat utilisation, this section proposes a hydrogen production system with a heat recovery system in multi-state operation.

### 2.1. Integrated System Structure

**Figure 1:**
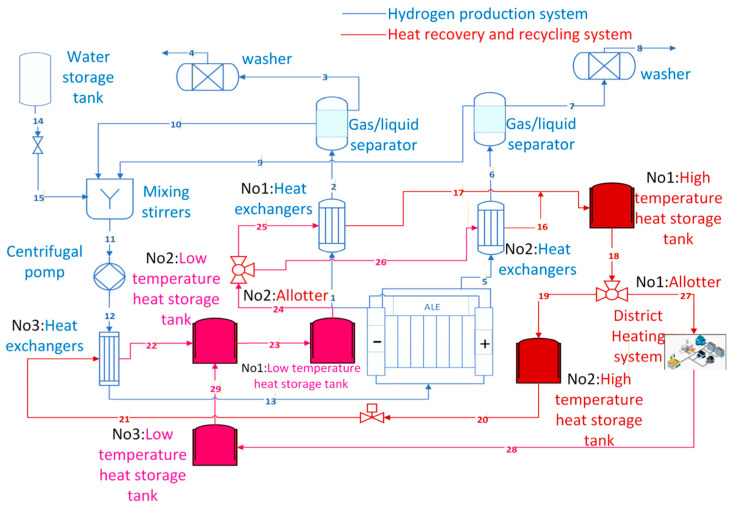
Structure of the hydrogen production system with heat recovery.

The hydrogen production system with a heat recovery cycle primarily consists of the hydrogen production system and the heat recovery cycle. As shown in Figure 1, the hydrogen production system comprises an AEC, a gas–liquid separator, a scrubber, a mixing agitator, and a centrifugal pump. The heat recovery cycle primarily consists of shell-and-tube heat exchangers and high- and low-temperature heat storage tanks.

The shell-and-tube heat exchangers (henceforth referred to as “heat exchangers”), designated No. 1 and No. 2 in the hydrogen production system, are positioned at the cathode and anode outlets of the AECs, respectively. This process reduces the overall temperature of the mixture of hydrogen, oxygen and electrolytes, thereby decreasing the water content in the hydrogen.

### 2.2. Thermodynamic Principles of the Heat Recovery Cycle

The thermodynamic principles of the heat recovery cycle have been established based on the principles of mass and energy conservation. A refined model of the heat recovery cycle system will be developed in Section 2.2. As shown in Table 1, m denotes the mass flow rate, h denotes the enthalpy, and T represents the temperature. j = 1,2,3 ……n denotes the corresponding number of the appliances shown in Figure 1. This paper hypothesises that the high-temperature heat storage tanks and the No. 2 and No. 3 low-temperature heat storage tanks can maintain a constant temperature for a limited period. The No. 1 heat storage tank has been observed to exhibit a heat dissipation effect, with unequal inlet and outlet temperatures.

### 2.3. Multi-State Operation Strategy of Alkaline Electrolysers

**Figure 2:**
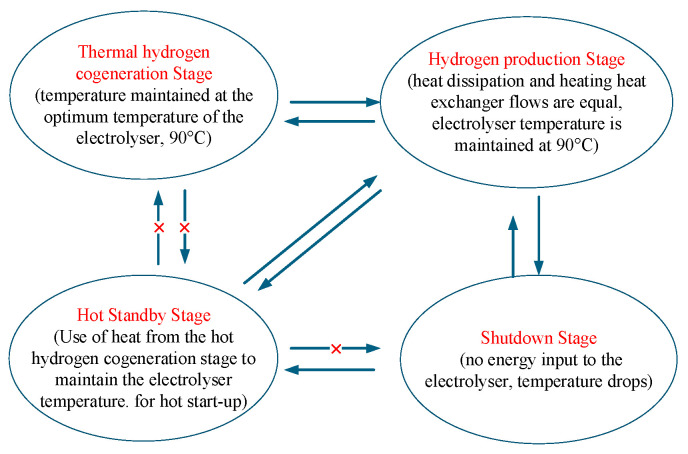
AECs operating states and transitions.

Based on the thermodynamic model of the heat recovery cycle, the working process of the AECs can be divided into four stages: the thermal hydrogen cogeneration stage, the hydrogen production stage, the hot standby stage, and the shut-down stage. As shown in Figure 2, during the hot hydrogen co-production stage, the input power reaches the rated value. The electrolytic cell’s heat generation exceeds its natural capacity for heat dissipation, and external equipment is required to maintain a lower temperature. In the hydrogen production stage, the input power is stable to achieve efficient hydrogen production. During the hot standby stage, when the input power is below the threshold, the electrolytic cell ceases to produce hydrogen. Still, it maintains its temperature through an external heat source, allowing for a quick restart. During the shut-down phase, low input power causes a temperature drop, and even after power is restored, the electrolytic cell will require a considerable amount of time to reheat to its operating temperature. We will optimise the conversion between each stage to reduce energy waste and equipment loss and improve the system’s overall performance.

This article considers the operating range of the AECs to be 30% to 100% of rated power, with an operating temperature range of 80 °C to 90 °C. When the input power is below 30%, the AECs stops running during the shut-down phase. Due to its heat dissipation, its temperature will gradually decrease. When the input power recovers to over 30% within the temperature range of 80 °C to 90 °C, the AECs can immediately return to the hydrogen production stage. As the duration for which the input power remains lower than 30% continues to lengthen, the temperature of the AECs drops below 80 °C. Even if the input power is restored to above 30%, the AECs still require a start-up time to reach the required temperature. Therefore, to shorten the start-up time, the AECs require an external heat source to maintain a temperature of 80 °C during the hot standby stage. When the electrical power input to the AECs is maintained at 90 °C, the temperature of the AECs will enter the hydrogen production stage if the heat generated by the electrolytic reaction is kept constant through effective heat dissipation. When the electrical energy input to the AECs continues to increase to the rated power, the heat generation of the AECs will exceed their heat dissipation. Currently, external devices are relied upon to cool the equipment and prevent damage resulting from excessive temperatures. This is the stage of hot hydrogen co-production.

## 3. Modelling of Hydrogen Production Systems with Heat Recovery

According to Section 2.3, the hydrogen production system with a heat recovery cycle comprises four working states of the AECs under the condition of renewable energy output. The previous simple hydrogen production system model can no longer meet the operating conditions of this multi-working condition, so in this section, a more detailed hydrogen production system model and heat recovery cycle model were established for modelling.

### 3.1. Hydrogen Production System Modelling

**Figure 3:**
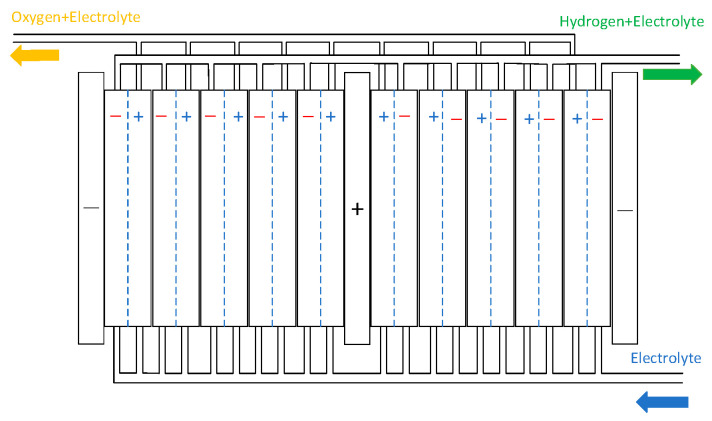
AECs structure.

To ensure targeted and feasible research, this study focuses on developing a detailed model of the electrolytic cell, which is fundamental to achieving the research objectives. The model of the gas–liquid separator can be found in [24]. The electrolytic cell is a bipolar structure composed of $N_{cell}$ electrolytic cells stacked together. The electrolytic cell features a symmetric configuration, with cathode electrodes positioned on both sides and an anode electrode centrally located. The anode chamber produces an alkaline solution and oxygen gas, while the cathode chambers generate hydrogen gas alongside an alkaline solution. The alkaline electrolyte is uniformly distributed from the bottom inlet through a circulation system and continuously flows back into both the anode and cathode chambers. The electrolysis stack of the AECs is shown in Figure 3 [25].

During the process of electrolysing water, an electrochemical reaction occurs, and water molecules are electrolysed into hydrogen molecules and oxygen molecules.

(1) $$\left\{\begin{array}{c} H_{2}O+\mathrm{electricity}+\mathrm{heat}=H_{2}+O_{2}\\ \mathrm{anodic}\;\mathrm{reaction}:\;4{\mathrm{OH}}^{-1}-4e^{-1}=O_{2}+2H_{2}O\\ \mathrm{cathodic}\;\mathrm{reaction}:\;4H^{+}+4e^{-}=2H_{2}\\ \mathrm{Total}\;\mathrm{energy}\;\mathrm{required}\;\mathrm{for}\;\mathrm{hydrogen}\;\mathrm{production}:\\ \Delta H=\Delta G+T\Delta S \end{array}\right.$$

where ∆H is the total energy required to decompose 1 mol of water molecules, ∆G is the Gibbs energy of water decomposition, *T* is the AECs temperature, and ∆S is the entropy change of water decomposition.

The following equation can express Gibbs free energy:

(2) $$\Delta G=U_{\mathrm{rev}}nF$$

where $U_{rev}$ is the reversible voltage, *n* is the number of electrons transferred to produce one mole of hydrogen, and *F* is the Faraday constant.

The enthalpy change (ΔH) can be expressed by the following equation:

(3) $$\Delta H=U_{\mathrm{th}}nF$$

where $U_{th}$ is the thermally neutral voltage.

To observe the energy consumption of the electrolytic cell under various operating conditions, this section presents the development of three interconnected models: energy consumption, efficiency, and temperature dynamics models.

#### 3.1.1. AECs Energy Consumption Modelling

The energy consumption index is the most critical indicator for evaluating AEC performance, which is closely related to the daily operating cost of the hydrogen production system. In this arrangement, the electrolytic chambers are segmented and interconnected through a series-parallel connection scheme. The summation of individual cell voltages determines the overall system voltage, each comprising a reversible voltage component and an irreversible voltage component. The irreversible voltage is further decomposed into three distinct overvoltage contributions: ohmic, activation, and concentration overvoltages. The reversible voltage is shown as follows [26]:

(4) $$U_{rev}=U_{rev}^{0}+\frac{RT}{nF}\ln\left(\frac{P_{H_{2}}P_{O_{2}}^{0.5}}{P_{H_{2}O}}\right)$$

where *R* is the ideal gas constant, and $P_{H_{2}},P_{O_{2}},P_{H_{2}O}$ represent the partial pressures of hydrogen, oxygen, and water, respectively.

The activation overvoltage is as folows [27]:

(5) $$\left\{\begin{array}{cc} U_{act} & =U_{act,a}+U_{act,c}\\ U_{act} & =\frac{RT}{\alpha_{a}nF}\ln\left(\frac{i}{i_{0,a}}\right)+\frac{RT}{\alpha_{c}nF}\ln\left(\frac{i}{i_{0,c}}\right)\\ \alpha_{a} & =0.1175+0.00095T\\ \alpha_{c} & =0.0675+0.00095T \end{array}\right.$$

where $U_{act,a}$ and $U_{act,c}$ denote the anode and cathode activation overvoltage, respectively, $\alpha_{a}$ and $\alpha_{c}$ denote the cathode and anode charge transfer coefficients, $i_{0,a}$ and $i_{0,c}$ denote the cathode and anode exchange current densities, and *i* denotes the current density.

The concentration overvoltage is

(6) $$U_{\mathrm{conc}}=-\frac{RT}{nF}\ln\left(1-\frac{i}{i_{L}}\right)$$

In: $i_{L}$ is the maximum current density.

The ohmic overpotential is

(7) $$U_{\mathrm{ohm}}=\left(2\rho_{\mathrm{electrode}}S_{\mathrm{electrode}}+\rho_{\mathrm{mem}}S_{\mathrm{mem}}+\rho_{\mathrm{electrolyte}}S_{\mathrm{electrolyte}}\right)i$$

(8) $$\rho_{electrode}=\frac{2}{\sigma_{Ni}}$$

(9) $$\sigma_{\mathrm{Ni}}=6,000,000-279,650T+532T^{2}-0.38057T^{3}$$

where $\rho_{electrode}$ is the resistivity of the electrode, $\delta_{electrode}$ is the thickness of the electrode, $\sigma_{Ni}$ is the conductivity of the electrode, $\rho_{mem}$ is the resistivity of the diaphragm, $\delta_{mem}$ is the thickness of the diaphragm, $\rho_{electrolyte}$ is the electrolyte resistivity, and $\delta_{electrolyte}$ is the electrolyte thickness.

The resistivity of the electrolyte is

(10) $$\left\{\begin{array}{c} \rho_{electrolyte}=\frac{1}{K}\\ K=(-2.041M-0.0028M^{2}+0.005332MT+207.7\frac{M}{T}+0.001043M^{3}-3\cdot 10^{-7}M^{2}T^{2})100\\ M=\frac{w\cdot \rho_{T}}{56.105\times 100}\\ \begin{array}{l} \rho_{T}=6.81w+1.137T+0.04391w^{2}+0.0138wT-0.002487T^{2}+0.0001279w^{3}-\\ 2.547\times 10^{-5}wT^{2}+868.1 \end{array} \end{array}\right.$$

where *K* is the conductivity of the electrolyte, *M* is the molar concentration, $\rho_{T}$ is the density of potassium hydroxide, and *w* is the concentration of potassium hydroxide.

The voltage of the electrolyser cell is

(11) $$U_{\mathrm{cell}}=U_{\mathrm{rev}}+U_{\mathrm{ohm}}+U_{\mathrm{conc}}+U_{\mathrm{act}}$$

The AEC energy consumption modelling is

(12) $$P_{AEC}=\frac{1}{2}U_{cell}IN_{cell}$$

#### 3.1.2. AEC Efficiency Model

Based on the AEC energy consumption model in Section 3.1.1, according to Faraday’s law, the hydrogen production rate is related to the electron transfer rate between the electrodes and the current. The generation rate of hydrogen and oxygen is proportional to the current, and the hydrogen production rate of a single electrolytic cell chamber is

(13) $${\dot{N}}_{H_{2}}=\frac{iA_{cell}}{2F}=2{\dot{N}}_{O_{2}}={\dot{N}}_{H_{2}O}$$

where $A_{cell}$ denotes the surface area of a single electrolysis cell, and $N_{O_{2}}$ denotes the rate of oxygen production.

The Faraday efficiency is

(14) $$\eta_{F}=\frac{U_{rev}}{U_{cell}}$$

The overall rate of hydrogen production is

(15) $$N_{H_{2},\mathrm{cell}}=\eta_{F}\frac{iA_{\mathrm{cell}}}{2F}N_{\mathrm{cell}}$$

where $N_{cell}$ denotes the number of electrolyser cells.

The efficiency of the AECs is

(16) $$\eta_{\mathrm{cell}}=1-\frac{U_{\mathrm{ohm}}+U_{\mathrm{conc}}+U_{\mathrm{act}}}{U_{\mathrm{cell}}}$$

**Figure 4:**
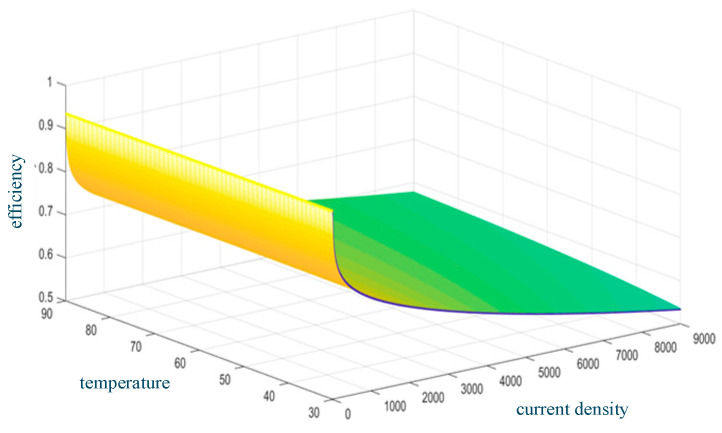
AEC efficiency versus temperature and current density.

At a constant temperature, the electrolysis efficiency decreases with the increase in current density. The electrolysis efficiency increases with temperature at a constant current density. Figure 4 shows the relationship between AEC efficiency, temperature, and current density.

#### 3.1.3. AECs Temperature Modelling

Figure 3 demonstrates the vital role of temperature in AEC operational characteristics. Elevated temperatures enhance solution conductivity, thereby improving the efficiency of the electrolysis process. Conversely, the magnitude of the electrolysis current directly impacts the operating temperature, creating a coupled thermal–electrical relationship that governs system efficiency. The temperature changes during the start-up and shut-down states of the AECs. Therefore, establishing an AEC temperature model is of great significance. This paper establishes the AEC temperature model using the lumped heat capacity method [28]. The heat dissipation model in this paper is established based on the work in Reference [29], and the model data are derived from our previous research finding [30].

(17) $$\begin{array}{cccc} & & \left\{\begin{array}{c} c_{t}\frac{dT}{dt}=Q_{gen}-Q_{liq}-Q_{gas}-Q_{sys}^{t}\\ Q_{gen}=U_{cell}\cdot i\cdot N_{cell}\\ Q_{gas}=N_{H_{2}}\int_{T_{0}}^{T}C_{p,H_{2}}dT+N_{O_{2}}\int_{T_{0}}^{T}C_{p,O_{2}}dT\\ \begin{array}{l} Q_{sys}^{t}=(T_{ele}^{t}-T_{0})\lambda_{ele}P_{ele}\\ \lambda_{ele}=\frac{A_{ele}}{R_{ele}} \end{array}\\ Q_{liq}=Q_{exch,1}+Q_{exch,2}-Q_{exch,3}-Q_{gas} \end{array}\right. & \end{array}$$

where $C_{t}$ denotes the heat capacity of AECs, and $Q_{gen}$ denotes the heat produced by the AEC itself. $Q_{liq}$ denotes the heat carried away by the lye, $Q_{sys}^{t}$ denotes the heat dissipated by the AECs, and $T_{ele}^{t}$ represents the operating temperature of the electrolyser. $C_{p,H_{2}},C_{p,O_{2}}$ denote the specific heat capacity of hydrogen and oxygen. $\lambda_{ele}$ denotes the heat dissipation coefficient of the electrolyser per unit capacity. $A_{ele}$ denotes the effective heat dissipation surface area per unit capacity. $R_{ele}$ denotes the thermal resistance per unit area (representing the insulation performance), and $P_{ele}$ denotes the installed capacity of the electrolyser. $T_{0}$ denotes the ambient temperature. $Q_{exch,1},\;Q_{exch,2},\;Q_{exch,3}$ denote the heat exchange capacity of heat exchangers 1, 2, and 3, respectively.

### 3.2. Heat Recovery Cycle Modelling

According to Section 2.1, the three shell-and-tube heat exchangers comprise the core equipment of the entire heat recovery system. Therefore, we focus on establishing a model for the heat exchangers with the thermal storage tank model detailed in [26]. In engineering applications, various types of heat exchangers are available, including shell-and-tube heat exchangers, plate heat exchangers, in-tube heat exchangers, and spiral heat exchangers. Compared to other heat exchangers, shell-and-tube heat exchangers offer significant advantages in terms of high heat exchange efficiency, adjustability, reduced heat loss, pressure resistance, and ease of maintenance. Therefore, this article chooses shell-and-tube heat exchangers to participate in the heat recovery cycle. In the current heat-recovery loop modelling, the pressure drop induced by the heat exchanger and the piping/valves, as well as the resulting circulation pump power consumption, are not explicitly considered and are treated in a simplified manner. Considering that existing thermal insulation technologies are very mature, the heat dissipation of the storage tanks is not considered in this paper.

#### 3.2.1. Modelling of Shell-and-Tube Heat Exchangers

**Figure 5:**
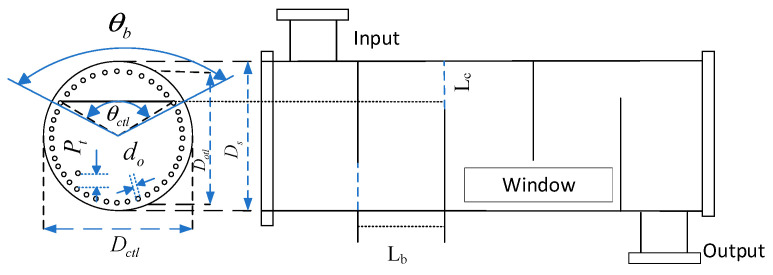
Geometry of shell-and-tube heat exchanger.

Firstly, the geometrical model of the shell-and-tube heat exchanger is established in Figure 5 [31]; $\theta_{b}$ is the outer angle at the folding plate, $\theta_{ctl}$ is the inner angle at the folding plate, $d_{o}$ is the outer diameter of the heat exchanger tubes, $P_{t}$ is the heat exchanger tubes’ centre spacing, $D_{ctl}$ is the outermost heat exchanger tube’s centre diameter, $D_{s}$ is the outer diameter of the shell, $L_{b}$ denotes the folding plate spacing, and $L_{c}$ denotes the center spacing from the folding plate’s opening to the tube’s inner wall. When considering the cost of the hydrogen production system equipment, in addition to the cost of the AECs, the equipment cost of the three shell-and-tube heat exchangers in the system should also be factored in. The equipment cost of shell-and-tube heat exchangers is closely related to the heat transfer area. In the subsequent optimisation of the shell-and-tube heat exchanger area, the above parameters not only describe the geometric characteristics of the heat exchanger but also serve as optimisation variables to optimise the heat exchanger area. The geometric structure modelling of shell-and-tube heat exchangers is detailed in Appendix A [32].

#### 3.2.2. Heat Transfer Modelling of Shell-and-Tube Heat Exchangers

In the hydrogen production system, the tube side of the three heat exchangers contains a mixture of electrolytes and gas, and the shell carries water as the working fluid. There was no phase transition between the working fluid on the tube and shell sides throughout the entire heat exchange stage. Without considering the heat dissipation of the heat exchanger, the first law of thermodynamics is as follows:

(18) $$Q=M_{h}\cdot C_{h}(T_{h,in}-T_{h,out})=M_{c}\cdot C_{c}(T_{c,in}-T_{c,out})U_{\mathrm{cell}}$$

where *Q* represents the heat exchange volume, $M_{h},\;M_{c}$ indicates the hot end and cold end mass flow of the work material, $C_{h},\;C_{c}$ represents the hot end and cold end of the heat capacity of the work material, and $T_{h,in},\;T_{h,out},\;T_{c,in},\;T_{c,out}$ represents the hot end of the import and export temperature of the work material and the cold end of the import and export temperature, respectively.

The heat transfer equation for the heat exchanger is [32]

(19) $$\left\{\begin{array}{c} Q=K_{ex}A\Delta t\\ \Delta t=\frac{\left(T_{h,\mathrm{in}}-T_{c,\mathrm{out}})-(T_{h,\mathrm{out}}-T_{c,\mathrm{in}}\right)}{\ln\left(\frac{T_{h,\mathrm{in}}-T_{c,\mathrm{out}}}{T_{h,\mathrm{out}}-T_{c,\mathrm{in}}}\right)} \end{array}\right.$$

where $K_{ex}$ is the heat transfer coefficient of the heat exchanger, *A* is the heat transfer area, and Δt is the average logarithmic temperature difference.

The heat transfer coefficient is

(20) $$K=1/(1/h_{i}\cdot d_{o}/D_{ti}+R_{ti}\cdot d_{o}/D_{ti}+R_{to}+delta_{wall}/K_{wall}\cdot d_{o}/(((d_{o}-D_{ti})/(ln(d_{o}))/D_{ti}))+1/h_{o}$$

In the formula, $h_{i}$ indicates the tube-side heat transfer coefficient; $d_{o}$ indicates the shell-side heat transfer coefficient, or the delta wall for the heat exchanger tube wall thickness; $D_{ti}$ indicates the heat exchanger tube’s inner diameter; $R_{ti}$, $R_{to}$ indicate the shell side of the fouling resistance; $K_{wall}$ indicates the thermal conductivity of the tube wall.

In the subsequent optimisation of heat recovery cycle parameters, a heat transfer efficiency model is established to prevent the heat exchanger area from approaching infinity [33]:

(21) $$\varepsilon =\frac{Q}{Q_{max}\frac{C_{h}\left(T_{h,i}-T_{h,o}\right)}{Ch,i_{c,i_{min}}\frac{C_{c}\left(T_{c,o}-T_{c,i}\right)}{Ch,i_{c,i_{min}}}}}$$

where ε is the heat transfer effectiveness of the heat exchanger, $Q_{max}$ is the maximum value of the ideal heat transfer of the heat exchanger, and $C_{min}$ represents the minimum value of the product of the mass flow rate and heat capacity of the work mass at the cold end and the hot end. According to [34], the tube-side heat transfer model of the shell-and-tube heat exchanger can be constructed (see Appendix B for details). According to [35], a shell-and-tube heat exchanger tube side heat transfer model is created (see Appendix C for more information).

## 4. Heat Recovery Cycle Design Optimisation Model

Based on the mathematical model of the core equipment of the system established in the previous text, this article optimises the parameter design of the heat recovery system under rated operating conditions, which can maximise energy efficiency and hydrogen production efficiency. Simultaneously, the equipment cost of shell-and-tube heat exchangers can impact the system’s economic viability. It is also necessary to ensure that the shell-and-tube heat exchanger has the lowest price at a specified mass flow rate and temperature. Thus, this section needs to carry out a two-stage optimisation. The first stage is to optimise the design parameters of the heat recovery cycle, and the second stage is to optimise the area of the heat exchanger.

### 4.1. Optimisation Model for Heat Recovery Cycle Design Parameters

The function of the heat recovery cycle is to maintain the optimal temperature of the AECs and maximise the recovery of waste heat from the system to supply the distributed system. After determining the operating parameters of the AECs, further optimising the operating parameters of the heat recovery cycle is beneficial for achieving the maximum waste heat recovery of the system while maintaining the temperature of AECs. Waste heat is used to supply distributed heating systems. Therefore, the first stage of optimisation uses the maximum exergy efficiency of the heat recovery system as the optimisation objective to optimise the operating parameters of the heat recovery cycle.

The hydrogen production rate under rated operating conditions is solely related to the AEC temperature, which is controlled by the thermal performance of the three heat exchangers. The three heat exchangers also determine the heat supplied to the distributed heating system. Referring to Figure 1, the unknown parameters for heat exchangers 1 and 2 include the tube-side outlet temperatures (ports 2 and 6), the shell-side inlet and outlet flow rates, and the shell-side outlet temperatures (ports 16 and 17). The inlet temperature on the shell side (ports 25 and 26) and the return water temperature of the distributed heating system (ports 28 and 29) are considered to be 25 °C. The inlet temperature of the tube side of heat exchanger 3 depends on the outlet temperatures of heat exchangers 1 and 2 (ports 2 and 6), so the unknown variables of heat exchanger 3 are the shell side outlet temperature and the shell side mass flow rate. To prevent the heat transfer area from tending towards infinity during the optimisation stage of shell-and-tube heat exchangers, the heat transfer efficiency of heat exchangers 1 and 2 is considered as an optimisation variable. In addition, the outlet temperature on the shell side of heat exchangers 1 and 2, as well as the outlet temperature on the shell side of heat exchanger 3, are also optimisation variables.

#### 4.1.1. Optimisation Objectives for Heat Recovery Cycle Parameters

For the three-heat-exchanger configuration, the inlet temperatures, outlet temperatures, and heat transfer effectiveness of units 1 and 2 are assumed to be equal. During optimisation under rated conditions, the thermal grade within the regenerative cycle is recognised as having a significant impact on system economics. Thermodynamic analysis under rated operating conditions reveals that the thermal grade distribution within the regenerative cycle has a considerable effect on the system’s techno–economic performance. Therefore, maximising exergy efficiency is established as the optimisation objective for the heat recovery system, as this criterion effectively integrates both thermodynamic performance and economic considerations into a single optimisation framework.

(22) $$F_{1}=\eta {\frac{\dot{E}X_{H_{2}}+\dot{E}X_{\mathrm{recover},H_{2}O}}{\dot{E}X_{\mathrm{elec}}}}_{\mathrm{exergy},\mathrm{design},\max}$$

Among them,

(23) $$\dot{E}X_{recover,H_{2}O}={\dot{m}}_{recover,H_{2}O}\cdot Cp_{H_{2}O}\cdot \Delta T\left(1-\frac{T_{0}}{T}\right)$$

(24) $$\dot{E}X_{\mathrm{elec}}=I\cdot U_{cell}$$

(25) $$\dot{E}X_{H_{2}}=\dot{E}X_{ph}+\dot{E}X_{ch}={\dot{N}}_{H_{2}}\left(\left({\bar{h}}_{i}-{\bar{h}}_{0})-T_{0}({\bar{s}}_{i}-{\bar{s}}_{0}\right)\right)+{\dot{N}}_{H_{2}}\bar{e}x_{ch,0}U_{cell}$$

where $\dot{E}X$ denotes the value of exergy, ${\overset{-}{h}}_{i}$ denotes the enthalpy of hydrogen at the temperature of the electrolyser; ${\overset{-}{h}}_{0}$ denotes the enthalpy of hydrogen at the reference temperature, ${\overset{-}{s}}_{i}$ denotes the entropy of hydrogen at the temperature of the electrolyser, and ${\overset{-}{s}}_{0}$ denotes the entropy of hydrogen at the reference temperature. $\overset{-}{e}{\overset{-}{x}}_{ch,0}$ denotes the standard chemical exergy. ${\dot{m}}_{re\mathrm{cov}er,H_{2}O}$ indicates that the 27 mass flow points in the system represent the heat supplied by the system to the distributed heating system, and ΔT denotes the temperature difference between the 21 ports and the 22 ports. *T* denotes the temperature of the electrolysis tank. *I* denotes the electrolysis current.

#### 4.1.2. Optimisation Constraints on Heat Recovery Cycle Parameters

Among the three heat exchangers, the first and second heat exchangers serve as heat dissipation devices, so the outlet temperature on the tube side should be higher than the inlet temperature on the shell side. The No. 3 heat exchanger acts as a heating element; therefore, the inlet temperature on the tube side should be lower than the outlet temperature on the shell side.

(26) $$T_{26}<T_{6},\;T_{25}<T_{2}$$

(27) $$T_{12}<T_{22}$$

A higher heat transfer effectiveness in a heat exchanger indicates greater heat exchange efficiency. However, as the effectiveness increases, the required heat transfer area also increases. When the effectiveness approaches 1, the heat transfer area theoretically approaches infinity. In practical applications, heat transfer effectiveness is generally maintained within the range of 0.85 to 0.95. The effectiveness values of heat exchangers 1 and 2 are designated as optimisation variables. Within the constraints, only the effectiveness of heat exchanger 3 is specified:

(28) $$0.85<effi_{NTU2}<0.95$$

### 4.2. Shell-and-Tube Heat Exchanger Heat Transfer Area Optimisation

The operating parameters of the heat recovery cycle are determined in the first stage. To ensure the optimal integration of the shell-and-tube heat exchangers with the overall system and minimise system costs, the structural parameters of the shell-and-tube heat exchangers need to be optimised at this stage.

#### Shell-and-Tube Heat Exchanger Area Optimisation Objective Function and Its Constraints

The shell-and-tube heat exchanger geometry is modelled in the Section titled: Shell and Tube Heat Exchanger Area Optimisation Objective Function and Its Constraints. The $d_{o}$, $D_{s}$ $P_{t}$, $L_{b}$, $L_{c}$ are selected as the optimisation variables, and the minimum heat exchanger area is taken as the optimisation objective:

(29) $$F_{2}=A_{\min}=\frac{Q}{K_{\mathrm{ex}}\Delta t}$$

The values of the aforementioned five optimisation variables are pivotal in determining the length of the shell-and-tube heat exchanger’s heat transfer tubes and the overall heat transfer coefficient. Consequently, this information is instrumental in ascertaining the area of the shell-and-tube heat exchanger. As demonstrated in [30], the following equation can be derived:

(30) $$0.3(m)<D_{s}<2(m)$$

(31) $$0.005(m)<d_{o}<0.05(m)$$

(32) $$1.15<\frac{P_{t}}{d_{o}}<2.5$$

(33) $$0.25<\frac{L_{c}}{D_{s}}<0.45$$

(34) $$0.3(m)<L_{b}<5(m)$$

(35) $$\frac{d_{o}}{D_{s}}<0.1$$

The presented methodology establishes a systematic optimisation framework for determining the optimal heat transfer area of shell-and-tube heat exchangers. This optimisation problem can be effectively solved through the implementation of the pattern search algorithm, utilising appropriate computational software platforms, which will be elaborated upon in Section 5.

## 5. An Interactive Optimisation Method for Hydrogen Production Systems with Heat Recovery Under Off-Design Conditions

The hydrogen production system with a heat recovery cycle involves multiple nonlinear components operating under different working conditions, which substantially increases operational complexity and amplifies nonlinear behaviour. Since AEC temperature and heat production fluctuate with variations in input current, this affects the working fluid mass flow rate on the shell side of the three shell-and-tube heat exchangers within the heat recovery circuit, as well as their heat exchange efficiency. The AEC temperature is controlled by these heat exchangers, which in turn affect AEC efficiency. When the system uses renewable energy for green hydrogen production, these subsystems interact dynamically, forming complex mutual influences.

**Figure 6:**
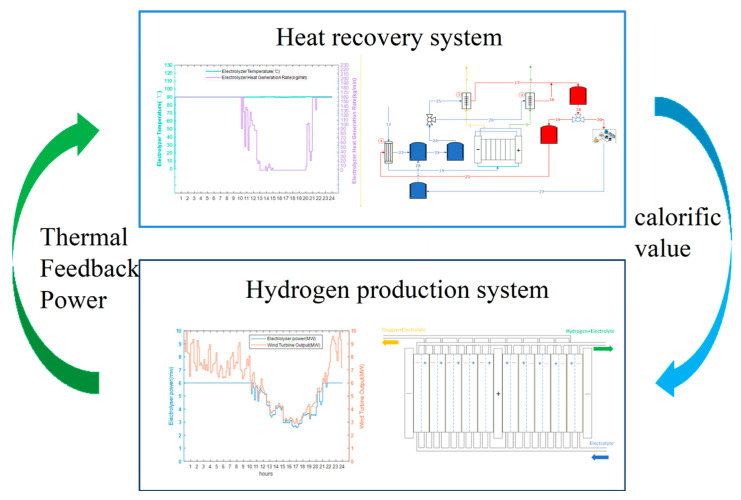
Schematic diagram of interactive optimisation of the hydrogen production system with heat recovery.

To address this challenge, this section proposes an interactive optimisation operation method for the integrated system under variable operating conditions. As illustrated in Figure 6, the approach uses the AEC temperature–efficiency characteristic curve as an Interactive Agent to optimise both the economic and system performance aspects of the heat recovery hydrogen production system.

### 5.1. Optimisation Objectives and Constraints for Variable Condition Scheduling of the Hydrogen Production System with Heat Recovery

Based on optimising the system parameters and heat exchange area of the heat recovery system under the rated operating conditions in Section 4, this section explores the overall optimisation scheduling of the system under variable operating conditions.

We choose maximising efficiency as the optimisation objective, and the optimisation variable is the input current of the electrolytic cell. In operation, the proposed scheme optimises the flow of heat transfer fluids to keep the hydrogen production system in an optimal state, even under varying operating conditions. The economic benefits of the integrated hydrogen production system with heat recovery are calculated by subtracting the operational costs from total revenue streams. Specifically, these costs encompass electricity consumption, alkaline electrolytic cell (AEC) operational expenses, and investments in heat exchanger equipment. Revenue is derived from hydrogen sales and the supply of thermal energy to the distributed heating system.

(36) $$\begin{array}{c} F=\max\left(\sum_{t=1}^{24}C_{H_{2}}M_{H_{2}}^{t}+C_{\mathrm{heat}}P_{\mathrm{heat}}^{t}-C_{\mathrm{elec}}P_{\mathrm{elec}}^{t}\right)-{\mathrm{cost}}_{ALE}-{\mathrm{cost}}_{exch} \end{array}$$

where F is the revenue of the system running for one day. $C_{H_{2}},C_{heat},C_{elec}$ denote the prices of hydrogen, heat, and electricity, respectively. $M_{H_{2}}^{t}$ denotes the production of hydrogen. $P_{heat}^{t},P_{ele}^{t}$ denote the heat power and electricity power, respectively. $\cos t_{ALE},\cos t_{exch}$ denote the cost of the AECs and heat exchanger, respectively.

A detailed model of AEC hydrogen production efficiency and hydrogen output has been established in the Section 3 and will not be further introduced here. The investment cost of AECs is the sum of investment cost and operating cost, and its model is

(37) $${\mathrm{cost}}_{\mathrm{ALE}}=\frac{\left(C_{\mathrm{invest}}P+C_{\mathrm{operation}}P\right)}{t_{\mathrm{operation}}}$$

where $C_{invest}$ indicates the unit price of AECs investment ($/KW), $C_{operation}$ indicates the unit price of AECs operation ($/KW), and $t_{operation\;}$ indicates the number of days of AECs operation.

The cost of the heat exchanger is its investment cost, which is modelled as [35,36]

(38) $$\left\{\begin{array}{cc} {\mathrm{cost}}_{\mathrm{exch}} & =\frac{C_{\mathrm{invest}}^{\mathrm{ex}}\cdot CEPCI_{2020}/15/325}{CEPCI_{2001}}\\ C_{\mathrm{invest}}^{\mathrm{ex}} & =C_{\mathrm{invest}}^{0}(B_{1}+B_{2}F_{p}F_{M})\\ \log_{10}C_{\mathrm{invest}}^{0} & =H_{1,\mathrm{exch}}+H_{2,\mathrm{exch}}\log_{10}(A_{\mathrm{exch}})+H_{3,\mathrm{exch}}{\left(\log_{10}(A_{\mathrm{exch}})\right)}^{2} \end{array}\right.$$

where $C_{invest}^{ex}$ is the annualised investment cost. *CEPCI* is the Chemical Engineering Plant Cost Index. $CEPCI_{2001}$ is chosen as the costing benchmark according to international thermal equipment costing practice, and *CEPCI*_2020_ is used as the inflation correction for the cost of thermal equipment, according to $CEPCI_{2020}$. In this study, CEPCI 2020 is adopted as the escalation reference because the equipment cost correlations and the assumed cost basis are referenced to the 2020 price level. If a more recent index is preferred, the investment costs can be readily updated by applying the ratio between the selected CEPCI value and CEPCI 2020. Such an update affects absolute cost values but does not alter the proposed methodological framework or the relative comparisons among the investigated cases. $B_{1},B_{2}$ are the price constant coefficients. $F_{p},F_{m}$ are the pressure correction coefficient and mass correction coefficient, where $H_{1,exch},\;H_{2,exch},\;H_{3,exch}$ are the cost-correlation coefficients for the shell-and-tube heat exchanger, adopted from [37]. The coefficient values are provided in Table 2.

The constraints imposed by optimising the system for variable operating conditions can be attributed to temperature and input power limitations.

(39) $$T<T_{\max}$$

(40) $$0<P_{\mathrm{ele}}^{t}<P_{AEC}$$

When the heat recovery cycle system operates under variable conditions, the mass flow rate of water on the shell side of the shell-and-tube heat exchanger must be dynamically adjusted based on the mass flow rate and temperature of the working fluid at the AECs anode and cathode outlets. The control strategy aims to maximise system efficiency while maintaining the outlet temperature as constant as possible. Under variable operating conditions, the mass flow rate of the AECs electrolyte remains a predetermined parameter established during the initial design phase of the electrolytic cell. The gas flow rate produced by electrolysis exhibits minimal deviation from the nominal flow rate designed under rated operating conditions. Consequently, the shell side outlet temperature of the shell-and-tube heat exchanger operates in constant temperature mode. The optimisation objective is to maximise the system’s exergy efficiency. The optimisation variables are the heat transfer efficiency of heat exchangers 1 and 2. In addition, consider the shell side outlet temperature of heat exchangers 1 and 2, as well as the shell side outlet temperature of heat exchanger 3, as optimisation variables. The optimisation objective is

(41) $$F_{3}=\eta {\frac{\dot{E}X_{H_{2}}+\dot{E}X_{\mathrm{recover},H_{2}O}}{\dot{E}X_{\mathrm{elec}}}}_{\mathrm{exergy},\max}$$

Constraints: The constraints in Equations (26) and (27) are the same. However, it is also necessary to consider the physical equipment constraints regarding whether the heat transfer area of the shell-and-tube heat exchanger under varying operating conditions is equal to that designed for the rated operating conditions.

(42) $$A_{\mathrm{ex}}=A$$

### 5.2. Interactive Optimisation-Based Solution for Operational Optimisation of the Hydrogen Production System Including Heat Recovery

Based on the temperature efficiency characteristic curve proposed in the appeal as an Interactive Agent, optimise the various operating parameters of the system under variable operating conditions. The specific optimisation steps are shown in Figure 6. When optimising, the first step is to determine the design parameters of the heat recovery system and the heat transfer area of the shell-and-tube heat exchanger in the hydrogen production system with heat recovery under rated operating conditions using the optimisation method provided in Section 3. The second step is to optimise the economic efficiency of the entire system under variable operating conditions, using an optimisation method for the hydrogen production system with heat recovery. Step three is to obtain the efficiency temperature curve based on the optimisation method of the hydrogen production system under variable operating conditions. Step four is based on receiving the curve shown above. Obtain the mass flow characteristic curve of the working fluid on the shell side of the heat recovery cycle based on the optimisation method for variable operating conditions of the heat recovery cycle. Based on the operating parameters of the heat exchanger and the optimisation of the structural parameters of the shell-and-tube heat exchanger, as mentioned in Section 4.2, the minimum heat transfer area under variable operating conditions is determined. Compare whether the heat transfer area of this heat recovery cycle system is equal to the designed heat transfer area under rated conditions. If not, change the input current to the AECs and recalculate until the developed area equals the optimised area at this time. Then, analyse whether the economy of the hydrogen production system with heat recovery has decreased. If the economy has reduced, reevaluate the economic type. If it has not decreased, the optimisation ends.

**Figure 7:**
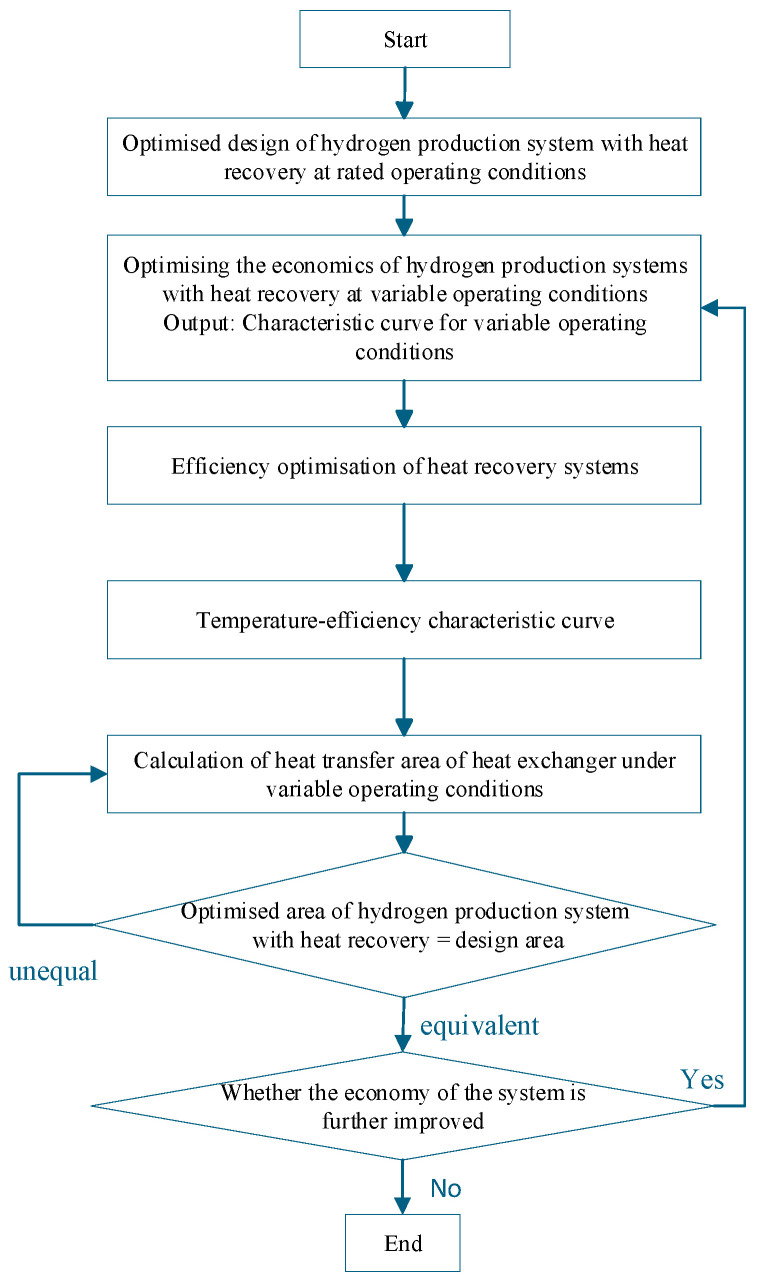
Calculation flow of the interactive optimisation method for variable operating conditions of the hydrogen production system with heat recovery.

Based on the temperature–efficiency characteristic curve serving as an Interactive Agent, the system’s operating parameters are optimised under variable operating conditions through an iterative algorithm, as shown in Figure 7. The process begins by determining the design parameters of the heat recovery system and the heat transfer area under rated conditions (Section 4). It then optimises the system’s economic efficiency under variable conditions to construct the temperature–efficiency curve and derive the shell-side working fluid mass flow characteristic curve. The minimum heat transfer area under variable conditions is subsequently determined and compared with the rated design area. If areas are unequal, the AEC input current is iteratively adjusted until they match. If economic performance deteriorates during this process, the optimisation returns to the economic efficiency step; otherwise, the optimisation is complete. This approach ensures optimal system performance while maintaining design consistency across operating conditions.

## 6. Illustrationive Case Study and Result

### 6.1. System Parameters

Qinghai has abundant wind and solar resources. To correspond to the installed capacity of the wind farm selected in this article, the calculation parameters are AECs with a rated power of 6 MW. The operating parameters of the AECs are shown in Table 3. Based on the optimisation model of the heat recovery system proposed in Section 4.1, a case study was constructed using data from a wind farm with an installed capacity of 10 MW in a specific area of Gonghe, Qinghai Province. The wind power data used in this study were obtained from the operational records of the Gonghe Wind Farm, operated by National Electric Group Of The Yellow River Upstream Hydropower Development Co., Ltd. (SPIC Qinghai Company, Xining, China). in April 2023. The system operational analysis considers three scenarios characterised by varying wind resource availability: an abundant wind resource scenario, a moderate wind resource scenario, and a scarce wind resource scenario. The system is simulated with a 1 h step and a 24 h cycle. The hydrogen price is set at 6.97 $/KG. The electricity price is 0.35 $/kW·h. Unlike other parameters derived from generalized literature, the heating price was determined by referencing actual market rates in representative regions. For instance, the heating price in Beijing is approximately 44.45 CNY/GJ, while the residential heating rate in Xining is about 5.51 CNY/m^2^/month. To derive a specific value for Gonghe County, we consulted the heating price associated with industrial waste heat recovery in Reference [38]. Taking into account Gonghe County’s lower regional economic level compared to the reference cities, as well as the inherent cost advantages of waste heat recovery, we applied a downward correction to these benchmarks. This resulted in a reasonable estimated price of 5.58 $/GJ. The AECs investment cost is 976.3 $/KW, and the AECs operation cost is 19.5 $/KW. The ambient temperature T_0_ is set to −20 °C to represent typical winter conditions in Qinghai Province, China, where our electrolyser-based hydrogen production system is located, and often operates at around −20 °C. Therefore, T_0_ =−20 °C is adopted as a practical cold-climate scenario rather than a universal standard reference condition.

### 6.2. Optimised Design for Rated Operating Conditions

#### 6.2.1. Results of the Optimisation of the Design Parameters of the Heat Recovery Cycle System

According to the optimisation model proposed in Section 4.1, the optimal rated parameters under the design conditions of the heat recovery cycle can be obtained, as shown in Table 4.

#### 6.2.2. Results of Optimisation of Geometrical Parameters of Shell-and-Tube Heat Exchangers

According to the optimisation method and conditions shown in Section 4.2, the minimum area can be obtained for different arrangements of heat exchange tubes. The optimisation results for the structural parameters of three shell-and-tube heat exchangers are presented in Table 5.

From the optimisation results, it can be seen that when the arrangement of heat exchange tubes is 90°, the heat exchange area is maximised. When the heat exchange tubes of heat exchangers 1, 2, and 3 are arranged at a 90° angle, the heat exchange areas are 1889.9 m^2^, 2244.2 m^2^, and 1055.8 m^2^, respectively.

### 6.3. Multi-Condition Operation Strategy

**Figure 8:**
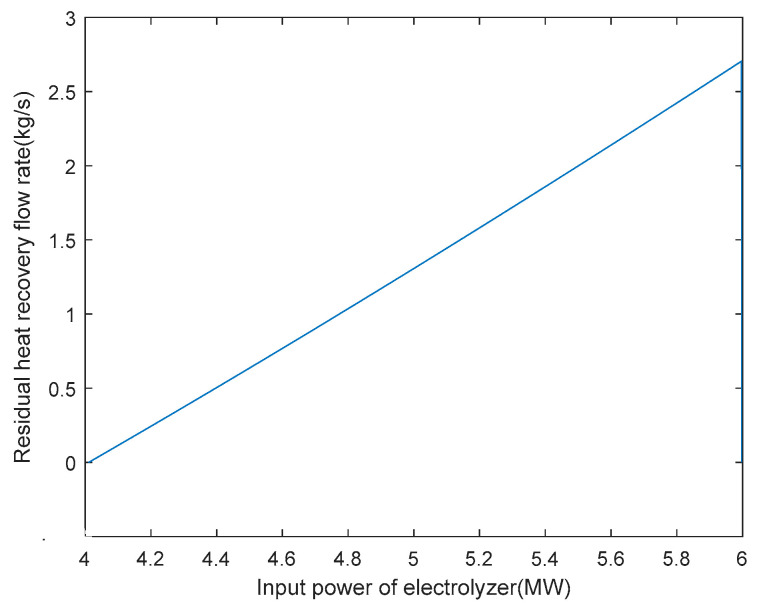
6 MW electrolyser heat recovery residual flow rate versus electrolyser input power.

Based on obtaining the optimal arrangement of heat exchange tubes and the heat exchange area of the heat exchanger under rated operating conditions, the optimisation of the hydrogen production system with heat recovery can be carried out under variable operating conditions. After determining the various parameters of the AECs, the relationship between the residual flow rate of 6 MW AEC heat recovery and the input power of the electrolytic cell can be defined under a certain input power, as shown in Figure 8. From the graph, it can be seen that when the power exceeds 4 MW, the heat generation of the electrolytic cell results in a heat recovery flow rate greater than 0. The AEC, with a rated power of 6 MW, is currently in the hot hydrogen co-production stage, transitioning from 4 MW to 6 MW of power. As mentioned previously, this article considers the operating range of AECs to be 30% to 100% of the rated power. Hence, the hydrogen production stage occurs when the input power is between 1.8 MW and 4 MW. When the input power is below 1.8 MW and the temperature is below 353.15 K, the system is in the hot standby stage. The shut-down phase occurs when the input power is below 1.8 MW and the temperature is between 353.15 K and 363.15 K.

To verify the economic feasibility of the proposed model and optimisation method, the following two options are compared.

Option 1: Adopt the hydrogen production system with heat recovery cycle proposed in this article. When the input power of the AECs is below 30% of the rated power and the temperature drops below the operating temperature range, the heat recovered by the heat recovery cycle is used to supply heat to AECs. The temperature of AECs is maintained at the lowest value of the operating temperature.

Option 2: Hydrogen production system without a heat recovery cycle. Use a cooling system to cool it down during the hydrogen production stage. When the input power of the AECs is below 30% of the rated power, it is shut down, and the AEC temperature continues to decrease.

#### 6.3.1. Analysis of Operational Simulation Results

- 1. Enriched wind resource scenarios.

**Figure 9:**
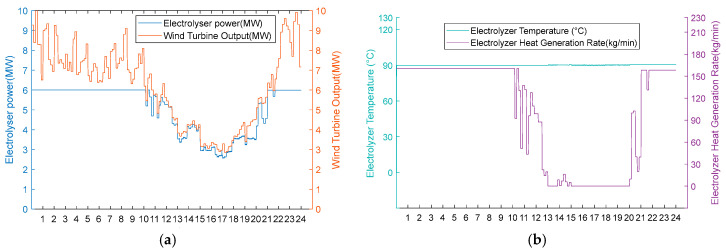
(**a**) In the abundant wind resource scenario, the turbine output, electrolyser power, and electrolyser operation status are considered. (**b**) Electrolyser temperature and residual flow rate for heat recovery from the electrolyser for the abundant wind resource scenario.

Under the abundant wind resource scenario, the AEC operational status is illustrated in Figure 9. During periods 0:00–13:00, 14:00–15:00, and 20:00–24:00, high wind turbine output results in wind power input exceeding 4 MW, enabling AEC operation in the thermal–hydrogen co-production mode (operational status colour coding is described in Section 5.1). In this mode, the recoverable heat from the heat recovery cycle increases in proportion to the input power. At the same time, the AEC temperature is maintained at 90 °C, simultaneously producing hydrogen and supplying heat to the distributed heating system. During intermediate periods (13:00–14:00 and 15:00–20:00), reduced wind turbine output results in the input power dropping below 4 MW but remaining above 3.6 MW, transitioning AEC to the hydrogen production mode. In this phase, the recoverable heat flow becomes zero, with the AECs dedicated solely to hydrogen production, operating without an external heat supply. Nevertheless, the electrolytic cell continues to generate excess waste heat, which is dissipated through the low-temperature heat storage tank No. 2, thereby maintaining the AEC temperature at 90 °C. Given the consistently abundant wind resources, the AEC input power remains above 1.8 MW throughout the operational period. Consequently, no operational difference exists between Scheme 1 and Scheme 2, resulting in equivalent economic performance for both configurations under this scenario.

- 2. Medium wind resource scenarios.

**Figure 10:**
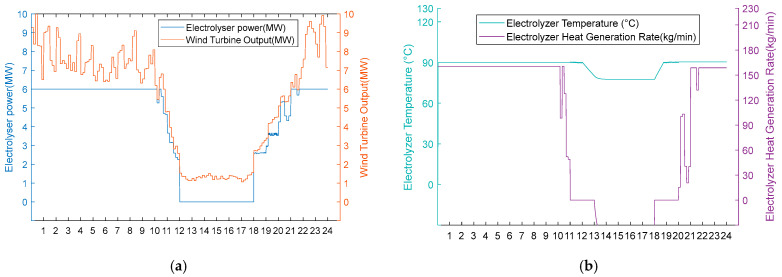
(**a**) Adoption of Option 1 turbine output, electrolyser power, and electrolyser operation status for the medium wind resource scenario. (**b**) Option 1 electrolyser temperature and residual flow rate for electrolyser heat recovery using the medium wind resource scenario.

**Figure 11:**
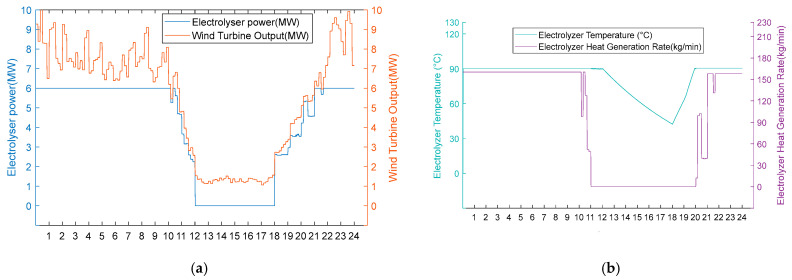
(**a**) Option 2: turbine output, electrolyser power, and electrolyser operation using the medium wind resource scenario. (**b**) Option 2: electrolyser temperature and residual flow rate for heat recovery from the electrolyser using Option 2 for the medium wind resource scenario.

Under moderate wind resource conditions, operational performance divergence occurs during wind deficiency periods, while maintaining identical behaviour during phases of sufficient wind availability. As shown in Figure 10a and Figure 11a, both schemes operate uniformly during high wind periods (0:00–11:00, 20:00–24:00) in thermal–hydrogen co-production mode and during moderate wind periods (11:00–12:00, 18:00–20:00) in hydrogen production mode. However, during the critical wind shortage period (12:00–18:00), Scheme 1 activates heat recovery thermal management, maintaining AEC operational readiness at 80 °C through heat exchanger No. 3 operation (50 kg/min shell-side flow, 84.98 °C inlet temperature, 35 °C outlet temperature) while optimising energy consumption by operating heat exchangers 1 and 2 at zero flow. Conversely, Scheme 2 undergoes passive thermal degradation at 42 °C due to the absence of thermal support infrastructure, as illustrated in Figure 10b and Figure 11b. Upon wind recovery at 18:00, Scheme 1 achieves immediate operational resumption. At the same time, Scheme 2 requires an approximately 90 min thermal recovery period, demonstrating the operational advantage of integrated heat recovery systems during moderate renewable energy intermittency.

- Option 1.

- Option 2.

- 3. Scarcity of wind resource scenarios

**Figure 12:**
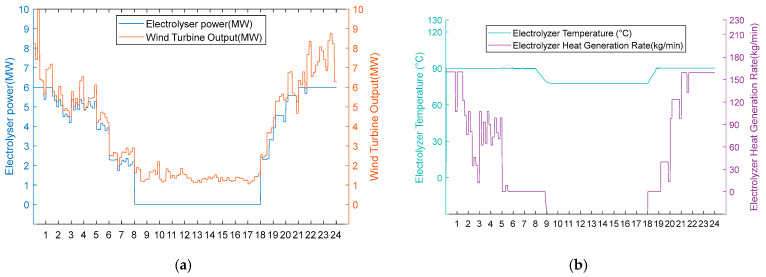
(**a**) Adoption of Option 1 wind turbine output, electrolyser power, and electrolyser operation status for the scarce wind resource scenario. (**b**) Adoption of Option 1 electrolyser temperature and residual flow rate for electrolyser heat recovery for the scarce wind resource scenario.

**Figure 13:**
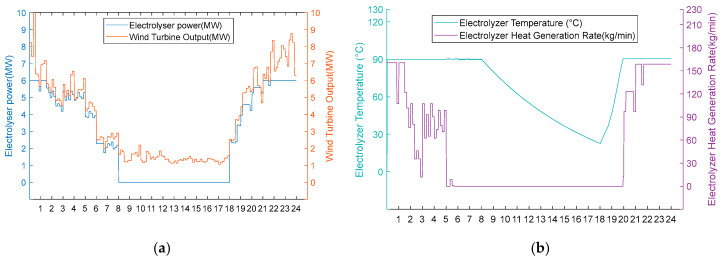
(**a**) Option 2: turbine output, electrolyser power, and electrolyser operation status using the scarce wind resource scenario. (**b**) Option 2: electrolyser temperature and residual flow rate for heat recovery from the electrolyser, using Option 2 for the scarce wind resource scenario.

Under conditions of scarce wind resources, operational performance differences also occur during periods of insufficient wind power. In Figure 12a and Figure 13a, it can be seen that these two schemes operate uniformly in hot hydrogen production mode during high wind periods (0:00–8:00, 18:00–24:00). However, the fan output continues to decrease from 8:00 to 18:00. The electrolytic cell is in the shut-down stage at this time. From Figure 12b, it can be seen that Scheme One reduces the temperature of the electrolytic cell to 80 °C from 8:00 to 9:00 when there is no energy input to the cell. From 9:00 to 18:00, the electrolytic cell is maintained at a temperature of 80 °C by the recovered heat. It immediately enters the hydrogen production stage when the fan output is restored at 18:00. From Figure 13b, it can be seen that the electrolytic cell in Scheme 2 is in a shut-down state from 8:00 to 18:00. Due to the lack of energy input, the temperature of the electrolytic cell continues to decrease to 22.6 °C. At 18:00, the fan output is restored. Due to the temperature not being within the operating temperature range, the electrolytic cell had a two-hour start-up period during which the temperature did not meet the standard, resulting in a zero hydrogen production rate. This proves that the heat recovery system eliminates the energy consumption and time cost associated with the cold start of the electrolytic cell by reusing waste heat.

- Option 1.

- Option 2.

#### 6.3.2. Analysis of Operational Strategies

To ensure the reproducibility of the economic results, the case study is conducted on a wind farm with an installed capacity of 10 MW in Gonghe, Qinghai Province, China, and the AEC rated power is set to 6 MW, with operating parameters listed in Table 3. The system is simulated over a 24 h horizon with a 1 h time step. The economic parameters used in this section are consistent with the parameter settings in Section 6.1, including a hydrogen selling price of 6.97 $/kg, an electricity price of 0.35 $/kWh, and a heating price of 5.58 $/GJ. The AEC investment cost and operation cost are set to 976.3 $/kW and 19.5 $/kW, respectively.

Table 6 shows the economic comparison between Option 1 and Option 2 in three scenarios. It is not difficult to see that there is no significant difference between Option 1 and Option 2 in the case of sufficient wind resources. However, under moderate wind resources and scarce wind resources, Option 1 increased its revenue by $512 and $737.70, respectively, compared to the results for Option 2 when electricity consumption is low. The hydrogen production rate of Option 1 has increased by 79.76 kg and 103.38 kg, respectively, compared to the results for Option 2. Under wind-scarce conditions, the proposed system with heat recovery produced 1211.9 kg of hydrogen, compared to 1108.52 kg in the baseline scenario without heat recovery. This represents a production increase of approximately 9.33% (calculated as (1211.9 − 1108.52)/1108.52 × 100%). It can be seen that the longer the wind power output is in a shortage scenario, the longer the shut-down time of the AECs. Option 2 will have a lower cost during AECs shut-down time, resulting in a longer start-up time and lower hydrogen production rate. At this point, Option 1 highlights its economic viability, significantly improving energy utilisation and system revenue. In addition, the updated Table 6 further quantifies the performance advantage of Option 1 over Option 2 in terms of energy-use metrics. Under medium wind resources and scarce wind resources, Option 1 reduces the specific electricity consumption by 2.54 and 5.06 kWh/kg-H_2_, respectively, compared with the results for Option 2 and achieves an absolute energy-efficiency improvement of 2.75% and 5.33%, respectively.

Under the same input of electrical energy, the hydrogen production device with an integrated heat recovery system can achieve an increase in hydrogen production at the thermodynamic level, recovering reaction waste heat, preheating reactants, reducing reaction activation energy, and ensuring stable operation of the system in the optimal temperature range. In terms of the economy, this technology reduces the unit cost of hydrogen and is significantly better than traditional systems. Research has shown that the heat recovery mechanism achieves a synergistic leap in hydrogen production, economy, and energy efficiency by converting previously ineffective waste heat into a reaction-driving force.

## 7. Conclusions and Future Research

### 7.1. Conclusions

Based on the traditional hydrogen production system with AECs [32], this paper introduces a heat recovery cycle to form a hydrogen production system with heat recovery. Under the condition of using new energy wind power as the power source, the multi-state operation of AECs is considered. The multi-state operation model of the electrolyser provides a theoretical basis for constructing a high-precision green hydrogen system by quantifying state correlations and revealing nonlinear mechanisms. On this basis, since interactive optimisation can achieve multi-objective optimisation in highly nonlinear systems compared with the results for traditional optimisation methods (in this paper, the temperature–efficiency curve is used as the Interactive Agent during optimisation), a heat recovery hydrogen production system considering AEC multi-state operation based on interactive optimisation is proposed. The research conclusion is only in the simulation stage and has not been verified by actual engineering. The feasibility of its large-scale application still needs to be explored. This study focuses on analysing the operational efficiency of AEC systems under wind power integration, without further exploring the impact of multi-energy coupling scenarios on system performance. The complexity of the research scenarios needs to be expanded. Through case analysis, the following conclusions are drawn:

(1) In the context of new energy, this study proposes, for the first time, an interactive optimisation theoretical framework for multi-state AEC operation.

(2) This study applies the heat recovery hydrogen production system and interactive optimisation method to the scenario of wind resource scarcity in the Qinghai region. The hydrogen production capacity and system efficiency of the hydrogen production system have significantly improved.

(3) When the output of wind resources is insufficient, to increase the economic efficiency of the system, adding new energy such as photovoltaics in subsequent optimisation is considered to achieve wind-solar complementarity, reduce the downtime of electrolytic cells, and stabilise system revenue.

### 7.2. Limitations and Future Research

(1) This work focuses on the interactive optimisation of a wind-powered hydrogen production system with a heat-recovery cycle, based on detailed electro–thermal modelling of the electrolyser and process-level modelling of the heat-recovery loop. However, the hydraulic pressure drop in the heat-recovery circuit and the associated pumping power consumption were not explicitly included in the current formulation, and the heat-recovery subsystem was assumed to maintain constant performance over time.

Under high electrolysis current conditions, heat generation in the electrolyser typically increases; to maintain the stack temperature within allowable limits, a higher coolant flow rate may be required, which can significantly increase the pressure drop and the associated parasitic pumping power, thereby reducing the net benefit of heat recovery. In addition, fluctuations in wind power output can subject the heat-recovery equipment to frequent thermal cycling, potentially accelerating degradation processes such as thermal fatigue and corrosion. Future work will incorporate flow-dependent pressure-drop correlations and pumping power calculations into the optimisation framework and introduce degradation-aware performance models calibrated through subsystem- and system-level experimental validation.

(2) This study is currently conducted at the modelling and simulation stage, and the integrated heat-recovery loop has not yet been experimentally validated. Nevertheless, the proposed framework is grounded in physics-based electro-thermal modelling of the electrolyser and standard heat-transfer relations. Key parameters are adopted from the literature and typical engineering ranges, enabling a quantitative assessment of the coupled electrolysis–thermal-management behaviour under wind power fluctuations. Importantly, our research group has already established an electrolyser-based hydrogen production and storage system, which provides a practical basis for staged validation. In the next stage, we will first validate the electrolyser’s dynamic performance and temperature response under fluctuating power inputs using the existing platform. We will then integrate a heat-recovery module for subsystem-level characterization and subsequently, system-level demonstration.

Potential implementation challenges include the intermittent nature of wind power, which can induce frequent thermal cycling and strengthen the control coupling between electrolyser temperature regulation and the heat-recovery loop. These challenges can be mitigated by incorporating thermal buffering and bypass control, together with advanced supervisory control strategies, to maintain stable stack temperature while satisfying practical operating constraints.

## Figures and Tables

**Table 1:** Mathematical modelling of the heat recovery cycle.

Appliances	Mathematical Model
Heat exchanger No. 1	${\dot{m}}_{1}={\dot{m}}_{2},{\dot{m}}_{25}={\dot{m}}_{17}$
	$h_{17}-h_{25}=h_{1}-h_{2}$
Heat exchanger No. 2	${\dot{m}}_{5}={\dot{m}}_{6}\;,{\dot{m}}_{26}={\dot{m}}_{16}$
	$h_{16}-h_{26}=h_{5}-h_{6}$
Heat exchanger No. 3	${\dot{m}}_{12}={\dot{m}}_{13},\;{\dot{m}}_{21}={\dot{m}}_{22}$
	$h_{21}-h_{22}=h_{13}-h_{12}$
No. 1 high-temperature heat storage tank	${\dot{m}}_{16}+{\dot{m}}_{17}={\dot{m}}_{18}$
	$T_{16}=T_{17}=T_{18}$
No. 2 high-temperature heat storage tank	${\dot{m}}_{19}={\dot{m}}_{20}$
	$T_{19}=T_{20}$
No. 1 low-temperature heat storage tank	${\dot{m}}_{23}={\dot{m}}_{24}$
No. 2 low-temperature heat storage tank	${\dot{m}}_{29}+{\dot{m}}_{22}={\dot{m}}_{23}$
	$h_{29}+h_{22}=h_{23}$
No. 3 low-temperature heat storage tank	${\dot{m}}_{28}={\dot{m}}_{29}$
	$T_{28}=T_{29}$
Distributor No. 1	${\dot{m}}_{19}+{\dot{m}}_{27}={\dot{m}}_{18}$
Distributor No. 2	${\dot{m}}_{25}+{\dot{m}}_{26}={\dot{m}}_{24}$

**Table 2:** Coefficients used in Equation (38) for the shell-and-tube heat exchanger.

Coefficient	Value
$H_{1,exch}$	4.0336
$H_{2,exch}$	0.2341
$H_{3,exch}$	0.0497

**Table 3:** AEC operating parameters.

Parametric	Numerical Value
Rated Power	6 MW
Operating Temperature	80 °C–90 °C
AEC Rated Current (DC)	9454 A
AEC Rated Voltage	635 V
AEC Electrode Diameter	1.84 m
AEC Length	5.85 m
Electrolyser Cell	326
Bipolar Plate Distance	0.01 m
Potassium Hydroxide Concentration (w)	30%
Ambient Temperature (T_0_)	−20 °C
Circulating Alkali Solution Flow Rate	19.677 (kg/s)
Anode Exchange Current Density i_0,a_	6.55 A/m^2^
Cathode Exchange Current Densityi_0,c_	0.577 A/m^2^
$\mathrm{Anode}\;\mathrm{Transfer}\;\mathrm{Coefficient}\;(\alpha_{a}$)	0.384
$\mathrm{Cathode}\;\mathrm{Transfer}\;\mathrm{Coefficient}\;(\alpha_{c}$)	0.434
Electrode Thickness	0.003 m
Electrolyte Thickness	0.0025 m
Diaphragm Thickness	0.0003 m

**Table 4:** Results of heat recovery cycle operating parameters.

Parameter Name	Results
$effi_{NTU,1},effi_{NTU2}$	0.94
$effi_{NTU2}$	0.88
$T_{16}=T_{17}=T_{18}=T_{19}=T_{20}=T_{21}=T_{27}$	84.98 (°C)
$T_{28}=T_{29}$	25 (°C)
$T_{22}$	35 (°C)
$T_{23}$	33.11 (°C)
${\dot{m}}_{16}$	8 (kg/s)
$T_{24}=T_{25}=T_{26}$	25 (°C)
$\eta_{exergy,max}$	0.64
$N_{H_{2},cell}$	0.03 (kg/s)
${\dot{m}}_{27}$	3.2563 (kg/s)

**Table 5:** Optimisation results of rated operating conditions of the shell-and-tube heat exchanger.

Heat Exchanger Number	Parameter Name	Heat Exchanger Tube Arrangement			
		30°	45°	60°	90°
One	Ds (m)	1.3292	0.81162	1.6352	0.69978
	Do (mm)	0.005	0.005	0.005	0.005
	Pt (mm)	0.0075	0.0075	0.0075	0.0075
	Lc (m)	0.51192	0.27388	0.71365	0.27127
	Lb,c (m)	1.0677	0.50981	1.1257	0.57297
	Area (m^2^)	4118.6	2576	6289.5	1889.9
Two	Ds (m)	1.5256	0.95238	1.7673	0.75925
	Do (mm)	0.005	0.005	0.005	0.005
	Pt (mm)	0.0075	0.0075	0.0075	0.0075
	Lc (m)	0.54817	0.36782	0.67933	0.23849
	Lb,c (m)	1.3169	0.57992	1.3868	0.67942
	Area (m^2^)	5459.1	3590.2	8105.8	2244.2
Three	Ds (m)	0.93526	0.61597	2	0.53114
	Do (mm)	0.005	0.005	0.005	0.005
	Pt (mm)	0.0075	0.0075	0.03	0.0075
	Lc (m)	0.24862	0.21906	0.79593	0.2068
	Lb,c (m)	0.88292	0.40467	5	0.44715
	Area (m^2^)	1996.4	1445.2	1086.9	1055.8

**Table 6:** Economics of Options 1 and 2 under three scenarios.

Parametric	Abundant Wind Resources	Medium Wind Resources		Scarcity of Wind Resources	
	Scene I (II)	Option 1	Option 2	Option 1	Option 2
Power consumption (mw/h)	7306.1	5818.7	5820	3910	3913
Profit ($)	6520	4842	4330	784.1	46.4
Hydrogen production (kg)	2264.4	1788.34	1708.58	1211.9	1108.52
Absolute energy efficiency improvement (%)	—	2.75	0	5.33	0
Reduction in specific electricity consumption (kWh/kg-*H*_2_)	—	2.54	0	5.06	

## Data Availability

The data presented in this study are available on request from the corresponding author.

## Abbreviations

The following abbreviations are used in this manuscript:

AECs	alkaline electrolysis cells
SOEC	solid oxide electrolyser cells
PEMC	proton exchange membrane electrolyser cells

## Appendix A

(A1) $$D_{\mathrm{otl}}=D_{s}-\delta_{bb}$$

(A2) $$D_{\mathrm{ctl}}=D_{\mathrm{otl}}-d_{o}$$

(A3) $$\theta_{b}=2\cos^{-1}\left(1-\frac{2L_{c}}{D_{s}}\right)$$

(A4) $$\theta_{\mathrm{ctl}}=2\cos^{-1}\left(\frac{D_{s}-2L_{c}}{D_{\mathrm{ctl}}}\right)$$

(A5) $$F_{w}=\frac{\theta_{\mathrm{ctl}}}{2}-\frac{\sin\theta_{\mathrm{ctl}}}{2\pi }$$

(A6) $$F_{c}=1-2F_{w}$$

(A7) $$F_{bp}=\frac{(D_{s}-D_{\mathrm{otl}})L_{b}}{A_{o,\mathrm{cr}}}$$

(A8) $$A_{o,\mathrm{cr}}=\left[D_{s}-D_{\mathrm{otl}}+\frac{D_{\mathrm{ctl}}}{X_{t}}(X_{t}-d_{0})\right]L_{b}$$

(A9) $$A_{o,\mathrm{tb}}=\frac{\pi }{4}\left[{\left(d_{0}-\delta_{tb}\right)}^{2}-d_{0}^{2}\right]N_{t}(1-F_{w})$$

(A10) $$A_{o,\mathrm{sb}}=\pi D_{s}\frac{\delta_{tb}}{2}\left(1-\frac{\theta_{b}}{2\pi }\right)$$

(A11) $$N_{t}=\frac{\pi }{4D_{\mathrm{ctl}}^{2}}\frac{1}{C_{t}p_{t}^{2}}$$

(A12) $$\begin{array}{l} \delta_{tb}=0.4\times 10^{-3} \end{array}$$

(A13) $$\begin{array}{l} \delta_{sb}=3.1\times 10^{-3}+0.004D_{s} \end{array}$$

where $\delta_{bb}$ denotes the gap between the shell and the outermost tube, $F_{w}$ denotes the proportion of tube bundles in the window, $F_{c}$ denotes the proportion of tube bundles in the cross-section, $F_{bp}$ denotes the area of the bypass flow, $A_{o,cr}$ denotes the area of the shell center cross, $A_{o,tb}$ denotes the area of the leakage between the tube and the manifold, $A_{o,sb}$ denotes the leakage area between the shell and the folding plate, $X_{t}$ denotes the transverse tube spacing, $\delta_{tb},\delta_{sb}$ denote the tube folding plate gap and shell folding plate gap, respectively, $N_{t}$ is the number of bundles, and $C_{t}$ denotes the bundle constant.

## Appendix B

### Modelling of Tube-Side Heat Transfer Coefficients for Shell-and-Tube Heat Exchangers

According to [34], the shell-and-tube heat exchanger tube-side heat transfer model is

(A14) $$D_{ti}=d_{o}-2\delta_{\mathrm{wall}}$$

(A15) $$u_{m,\mathrm{tube},t}=\frac{M_{t}}{A_{o,t}}$$

(A16) $$\begin{array}{l} Re_{t}=u_{m,\mathrm{tube},t}\times \frac{D_{ti}}{{\mathrm{vis}}_{t}} \end{array}$$

(A17) $$\begin{array}{l} Pr_{t}={\mathrm{vis}}_{t}\times \frac{C_{pt}}{k_{t}} \end{array}$$

(A18) $$\begin{array}{l} f_{t}=0.79\times \log({\left(Re_{t}-1.64\right)}^{-2}) \end{array}$$

(A19) $$\begin{array}{l} h_{t}=\frac{\frac{k_{t}}{D_{ti}}\left(\frac{f_{t}}{8}(Re_{t}-1000)\cdot Pr_{t}\right)}{\left(1+12.7{\left(\frac{f_{t}}{8}\right)}^{0.5}\left(Pr_{t}^{2/3}-1\right)\right)} \end{array}$$

(A20) $$\begin{array}{l} h_{i}=h_{t}\cdot J_{c}\cdot J_{l} \end{array}$$

(A21) $$\begin{array}{l} J_{l}=0.44(1-r_{s})+\left(1-0.44(1-r_{s})\right)e^{-2.2r_{lm}} \end{array}$$

(A22) $$\begin{array}{l} J_{c}=0.55+0.72F_{c} \end{array}$$

(A23) $$\begin{array}{l} r_{s}=\frac{A_{o,\mathrm{sb}}}{A_{o,\mathrm{sb}}+A_{o,\mathrm{tb}}} \end{array}$$

(A24) $$\begin{array}{l} r_{lm}=\frac{A_{o,\mathrm{sb}}+A_{o,\mathrm{tb}}}{A_{o,\mathrm{cr}}} \end{array}$$

where $D_{ti}$ is the inner diameter of the heat exchanger tube, $A_{ot}$ is the flow area of each tube, $n_{tube,pass}$ denotes the tube length, $u_{m.tube,t}$ is the tube-side mass flow rate, $vis_{t}$ is the tube-side dynamic viscosity of the work mass, ${Re}_{t}$ is the tube-side Reynolds number, ${\mathrm{Pr}}_{t}\;$ is the tube-side Planck number, $Cp_{t}$ is the specific heat capacity of the work material on the tube side, $K_{t}$ is the heat transfer coefficient of the work material on the tube side, $f_{t}$ is the friction coefficient on the tube side, $h_{t}$ is the ideal heat transfer coefficient on the tube side, and $J_{l},J_{b}$ are the correction factors.

## Appendix C

### Modelling of Shell-Side Heat Transfer Coefficients for Shell-and-Tube Heat Exchangers

According to [35], the shell-and-tube heat exchanger tube-side heat transfer model is

(A25) $$h_{o}=h_{id,shell}\cdot J_{c}\cdot J_{l}\cdot J_{b}$$

where $h_{o}$ is the shell-side heat transfer coefficient, $h_{id,shell}$ is the shell-side ideal heat transfer coefficient, and $J_{b}$ is the correction factor ($J_{b}$ = 0.9).

The shell-side ideal heat transfer coefficient significantly influences the shell-side heat transfer coefficient. It is imperative to consider the impact of the arrangement between the tube bundles on the heat transfer area. The arrangements most commonly utilised in the project are 30°, 45°, 60°, and 90°. Among them, 30°, 45°, and 60° can use the same calculation model [35].

The ideal heat transfer coefficient model for the shell side when using 30°, 45°, and 60° tube arrangements is

(A26) $$h_{\mathrm{id}}=\frac{k}{D_{h}}0.404L_{q}^{1/3}$$

(A27) $$L_{q}=\left\{\begin{array}{cc} 0.92H_{g}Pr\left(\frac{4X_{t}^{*}}{\pi }-1\right)\frac{1}{X_{d}^{*}}, & X_{l}^{*}\geq 1,\\ 0.92H_{g}Pr\left(\frac{4X_{t}^{*}X_{l}^{*}}{\pi }-1\right)\frac{1}{X_{l}^{*}X_{d}^{*}}, & X_{l}^{*}<1. \end{array}\right.$$

(A28) Hglam=140Redx1∗0.5−0.62+0.75x1∗1.64xt∗x1∗π−1,X1∗≥0.52xt∗2+10.5140Redx1∗0.5−0.62+0.75xd∗1.64xt∗x1∗π−1,X1∗<0.52xt∗2+10.5

(A29) $$H_{\mathrm{gturb},s}=\left[1.25+\frac{0.6}{{\left(x_{t}^{*}-0.85\right)}^{1.08}}\right]+0.2{\left(\frac{x_{l}^{*}}{x_{t}^{*}}-1\right)}^{3}-0.005{\left(\frac{x_{t}^{*}}{x_{l}^{*}}-1\right)}^{3}Re_{d}^{1.75}.$$

(A30) $$H_{g}=H_{\mathrm{glam}}+H_{\mathrm{gturb},i}\left[1-\exp\left(1-\frac{Re_{d}+1000}{2000}\right)\right].$$

(A31) $$X_{d}=\sqrt{X_{l}^{2}+X_{t}^{2}},\;X_{d}^{*}=\frac{X_{d}}{d_{0}}.$$

The ideal heat transfer coefficient model for the shell side when using a 90° tube arrangement is

(A32) hid=kDh0.404Lq1/3Red+1Red+10000.1

(A33) $$D_{h}=\frac{1.27}{d_{0}}\left(p_{t}^{2}-0.875d_{0}^{2}\right)$$

(A34) $$L_{q}=1.18H_{g}\mathrm{Pr}\left(\frac{4X_{t}^{*}}{\pi }-1\right)\frac{1}{X_{l}^{*}}$$

(A35) $$X_{t}^{*}=\frac{X_{t}}{d_{0}}$$

(A36) $$X_{l}^{*}=\frac{X_{l}}{d_{0}}$$

(A37) Hglam=140RedXl∗0.5−0.62+0.75Xl∗1.64Xt∗Xl∗π−1

(A38) Hgturb,i=0.11+0.61−0.94Xl*0.6Xt*−0.851.3+0.015Xt*−1Xl*−1×Red2−0.1Xl*Xt*−1.5

(A39) Hg=Hglam+Hgturb,i1−exp1−Red+10002000

where *k* denotes the heat transfer coefficient of the fluid, *D* denotes the hydraulic diameter, $L_{q}$ indicates the Lévêque number, $H_{g}$ denotes the Hagen number, $P_{r}$ denotes the Prandtl number, $R_{ed}$ denotes the Reynolds number, $X_{t}$ denotes the transverse tube spacing, $X_{I}$ denotes the longitudinal tube spacing, $C_{t}$ denotes the tube bundle constant, and $X_{t}^{*},X_{l}^{*}$ are dimensionless constants which can be obtained from $X_{t}$ and $X_{l}$.

The values of the tube bundle constants in Equations (A10) and (A33), as well as the transverse tube spacing and the longitudinal tube spacing, are related to the tube arrangement, as shown in Table A1.

**Table A1:** Values of transverse and longitudinal tube spacing and tube bundle constants for different heat exchanger tube arrangements.

Heat Exchanger Tube Arrangement				
	30°	45°	60°	90°
X_t_ (transverse pipe spacing)	$P_{t}$	$\sqrt{2}P_{t}$	$\sqrt{3}P_{t}$	$P_{t}$
X_l_ (longitudinal pipe spacing)	$\frac{\sqrt{3}P_{t}}{2}$	$\frac{P_{t}}{\sqrt{2}}$	$\frac{P_{t}}{2}$	$P_{t}$
C_t_ (bundle constant (math))	$\frac{\sqrt{3}}{2}$	1	$\frac{\sqrt{3}}{2}$	1
